# Annexin A1 down-regulation in head and neck squamous cell carcinoma is mediated via transcriptional control with direct involvement of miR-196a/b

**DOI:** 10.1038/s41598-017-07169-w

**Published:** 2017-07-28

**Authors:** Saúl Álvarez-Teijeiro, Sofía T. Menéndez, M. Ángeles Villaronga, Emma Pena-Alonso, Juan P. Rodrigo, Reginald O. Morgan, Rocío Granda-Díaz, Cecilia Salom, M. Pilar Fernandez, Juana M. García-Pedrero

**Affiliations:** 1 Department of Otolaryngology, Hospital Universitario Central de Asturias and Instituto Universitario de Oncología del Principado de Asturias, University of Oviedo, Oviedo, CIBERONC Spain; 2 Department of Biochemistry and Molecular Biology, University of Oviedo, Oviedo, Spain

## Abstract

Annexin A1 (ANXA1) down-regulation is an early and frequent event in the development of head and neck squamous cell carcinomas (HNSCC). In an attempt to identify the underlying mechanisms of reduced ANXA1 protein expression, this study investigated ANXA1 mRNA expression in HNSCC specimens by both *in situ* hybridization and RT-qPCR. Results showed a perfect concordance between the pattern of ANXA1 mRNA and protein detected by immunofluorescence in tumors, precancerous lesions and normal epithelia, reflecting that ANXA1 down-regulation occurs at transcriptional level. We also found that both miR-196a and miR-196b levels inversely correlated with ANXA1 mRNA levels in paired HNSCC tissue samples and patient-matched normal mucosa. In addition, endogenous levels of ANXA1 mRNA and protein were consistently and significantly down-regulated upon miR-196a and miR-196b over-expression in various HNSCC-derived cell lines. The direct interaction of both mature miR-196a and miR-196b was further confirmed by transfection with *Anxa1* 3′UTR constructs. Combined bioinformatics and functional analysis of *ANXA1* promoter activity contributed to identify key regions and potential mediators of *ANXA1* transcriptional control. This study unveils that, in addition to miR-196a, miR-196b also directly targets ANXA1 in HNSCC.

## Introduction

Annexin A1 (ANXA1, also known as lipocortin 1 and calpactin II) is a pleiotropic 37 kDa protein member of the annexin superfamily with roles in vesicular trafficking and the maintenance of membrane-cytoskeletal integrity^1^. ANXA1 is considered a primary mediator of the anti-inflammatory, immunosuppressive actions of glucocorticoids^2^ and current attention has focused on its roles in membrane remodeling and cell behavior associated with regulatory signaling via the Formyl Peptide Receptor^3^ and oncogenesis^4^. These studies point to a major regulatory role for ANXA1 in cell-growth regulation and differentiation, neutrophil migration, CNS responses to cytokines, neuroendocrine secretion and apoptosis. The protein can be found in alternatively spliced isoforms, as proteolytic fragments of its bioactive N-terminus and as a heterotetramer with S100A11, localized to the nucleus, cytoplasm, membrane or extracellular matrix. It is a principal substrate for epidermal and hepatic growth factor (EGF and HGF) receptor tyrosine kinases and protein kinase C^5, 6^. These phosphorylation events target the PI3 kinase and ERK MAP kinase glucocorticoid signaling pathways to regulate cell proliferation, differentiation and apoptosis^7^. ANXA1 and other annexins are frequently deregulated in many cancers. ANXA1 overexpression has been reported in gastric^8^, pancreatic and hepatocellular carcinoma^9^ but it is markedly down-regulated in breast^10, 11^, prostate^12^, esophageal^13^ and head and neck squamous cell carcinomas^14^. The contrasting patterns of ANXA1 expression in different tumors types is just one of the enigmas in deciphering the underlying regulatory mechanisms and phenotypic specificity of ANXA1.

It is now well established that ANXA1 down-regulation can be detected at the protein level in early stage tumorigenesis of HNSCC tissue specimens and visual microscopic evidence confirms that this is significantly correlated with advanced disease stage, lymph node metastasis and poor tissue differentiation^14^. More recently, the marked reduction of ANXA1 expression in epithelial dysplasia and strong positive expression in differentiated layers of normal epithelia corresponded to inverse changes in levels of miR-196a under both conditions^15–18^.

Little is known about the underlying mechanisms responsible for the loss of ANXA1 expression in this type of cancer. Plausible mechanisms include genomic deletions, regulatory or coding mutations of the *ANXA1* gene, epigenetic silencing by promoter hypermethylation, miRNA-mediated repression, alteration of one or more proteins that regulate ANXA1 transcription such as IL-6, alterations in post-translational processing of the protein by proteolysis or phosphorylation, and defects of intracellular transport or protein localization that lead to reduced intracellular levels of ANXA1.

In an attempt to identify the cause of reduced ANXA1 protein expression in HNSCC, this study determined ANXA1 mRNA expression levels in HNSCC tissue specimens by both RT-qPCR and *in situ* hybridization. Since the ANXA1 3′UTR is a potential target of various microRNAs (15, 19 – miRBase, 20 – TargetScan) we focused on its documented regulatory relationship with the miR-196 family. These are noncoding RNAs derived from loci in the homeobox (HOX) gene clusters^21^ in human chromosomes 17q21.32 (MIR196A1), 12q13.13 (MIR196A2) and 7p15.2 (MIR196B), among which the mature microRNAs miR-196a1 and a2 are identical and miR-196b differs by one nucleotide. As individual microRNAs can regulate multiple genes^17, 18^ including those encoding transcription factors, we also investigated the potential contribution of transcriptional control by analysis of *ANXA1* promoter activity.

## Results

### Down-regulation of ANXA1 expression in HNSCC occurs at transcriptional level

**Figure 1:**
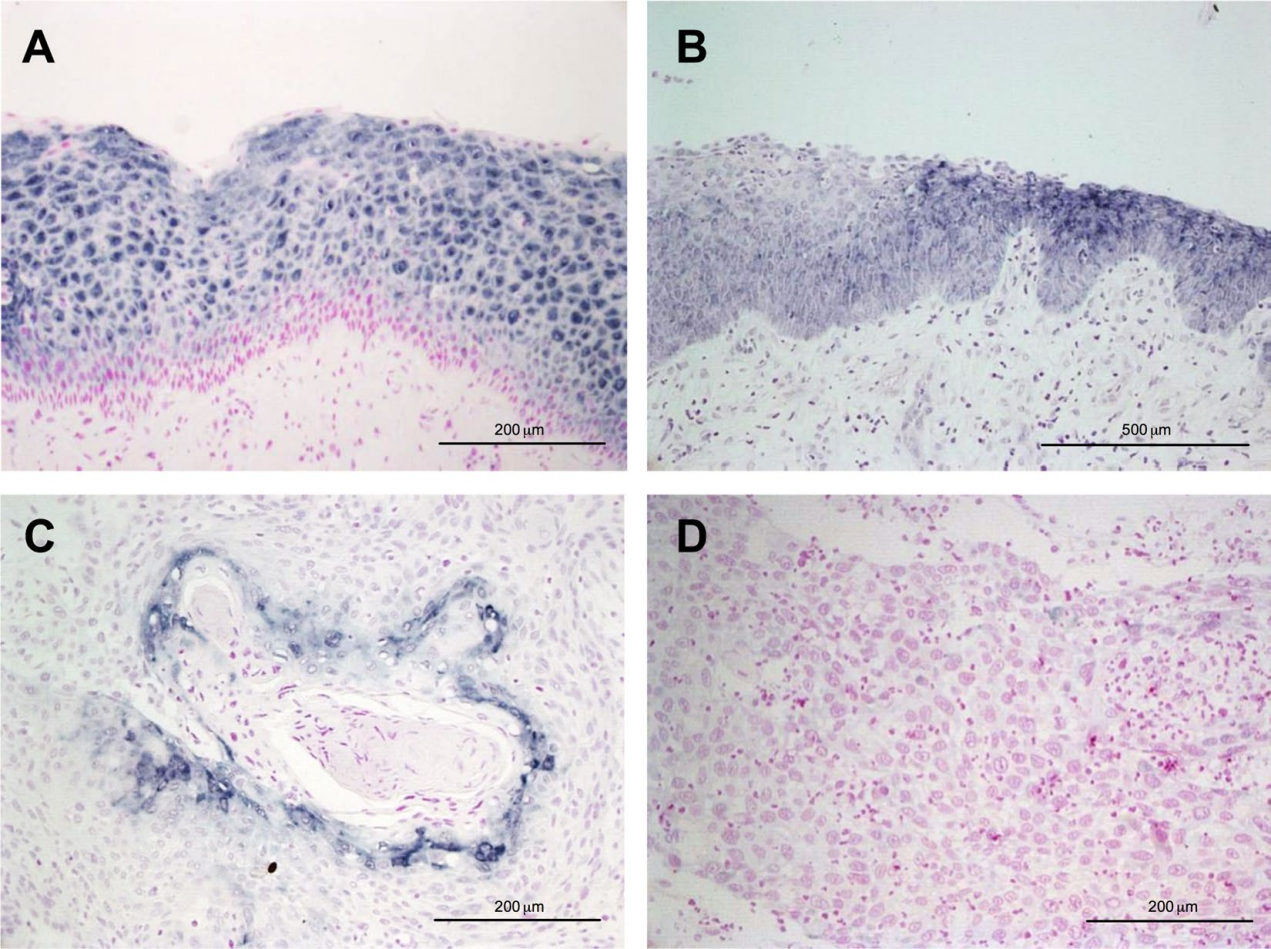
*In situ* hybridization analysis of ANXA1 mRNA expression (blue, contrasted with nuclear red stain) in normal epithelia (**A**), epithelial dysplasia (**B**), well-differentiated HNSCC (**C**), and poorly differentiated HNSCC (**D**). Magnification 20x (**A**,**C**,**D**), and 10x (**B**). Normal cells stain for ANXA1 in the superficial stratum, differentiated HNSCC exhibit positive mRNA expression in highly keratinized areas, and poorly differentiated HNSCC do not stain for ANXA1.

**Figure 2:**
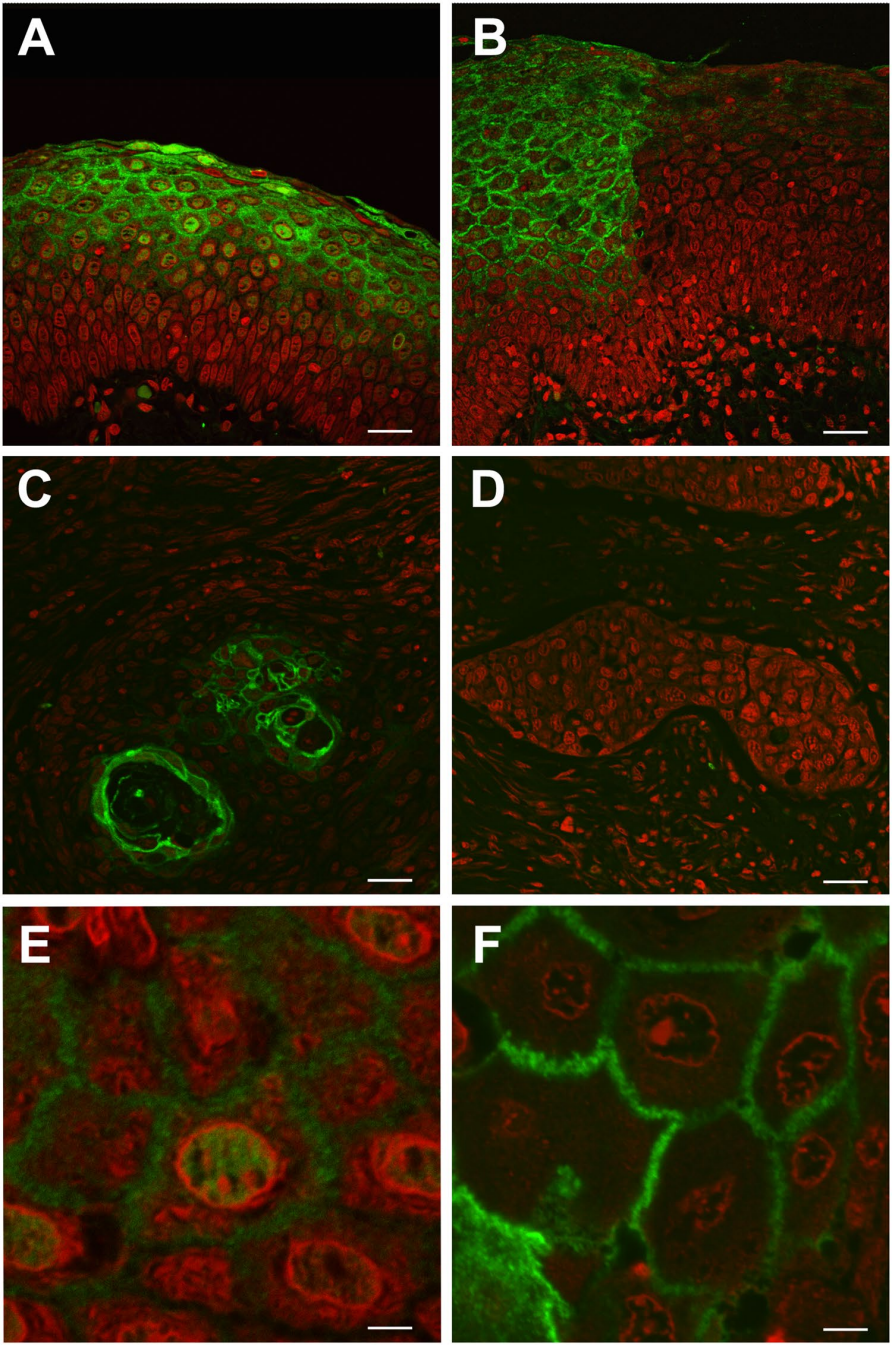
Immunofluorescence analysis of ANXA1 protein expression in HNSCC. Localization of ANXA1 protein (green) contrasted with propidium iodide (red) in normal epithelia (**A**), in epithelial dysplasia (*middle right*, **B**) and foci of differentiated HNSCC (**C**), and in poorly differentiated HNSCC (**D**). ANXA1 expression is restricted to the superficial stratus of well-differentiated cells in normal tissue (**A**) and localized to the cytoplasm, plasma membrane and nucleoplasm (excluding the nuclear membrane and nucleolus) of normal cells (**E**). In well-differentiated HNSCC, ANXA1 expression is markedly subdued and further restricted to the cornified envelope (**C**) where it is localized exclusively to the plasma membrane of remaining differentiated cells (**F**). ANXA1 expression is completely lost in poorly differentiated HNSCC (**D**). Scale bar 25 µm (**A**–**D**), and 5 µm (**E**,**F**).

We have previously reported that ANXA1 protein expression is frequently down-regulated in HNSCC tissue specimens, while highly expressed in the differentiated layers of normal epithelium^14^. ANXA1 expression at the mRNA level was assessed by *in situ* hybridization to investigate the possible underlying mechanisms of ANXA1 down-regulation in HNSCC. As shown in Fig. 1, the patterns of ANXA1 mRNA and protein expression (Fig. 2) perfectly matched. Strong ANXA1 mRNA expression was detected in the differentiated layers of normal epithelia while absent in basal and suprabasal cells (Fig. 1A). *In situ* hybridization confirmed the loss of ANXA1 mRNA expression in dysplastic epithelia (Fig. 1B) compared to normal adjacent epithelium. A general down-regulation of ANXA1 mRNA expression was observed in tumors. Well-differentiated HNSCC exhibited strong positive ANXA1 expression in highly keratinized areas (Fig. 1C), whereas poorly differentiated tumors showed negative ANXA1 expression (Fig. 1D). Identical results were obtained by immunofluorescence analysis of ANXA1 protein (Fig. 2A–F), demonstrating that ANXA1 down-regulation in HNSCC occurs at the transcriptional level.

### ANXA1 mRNA expression inversely correlated with miR-196a and miR-196b levels in HNSCC tissue specimens

**Figure 3:**
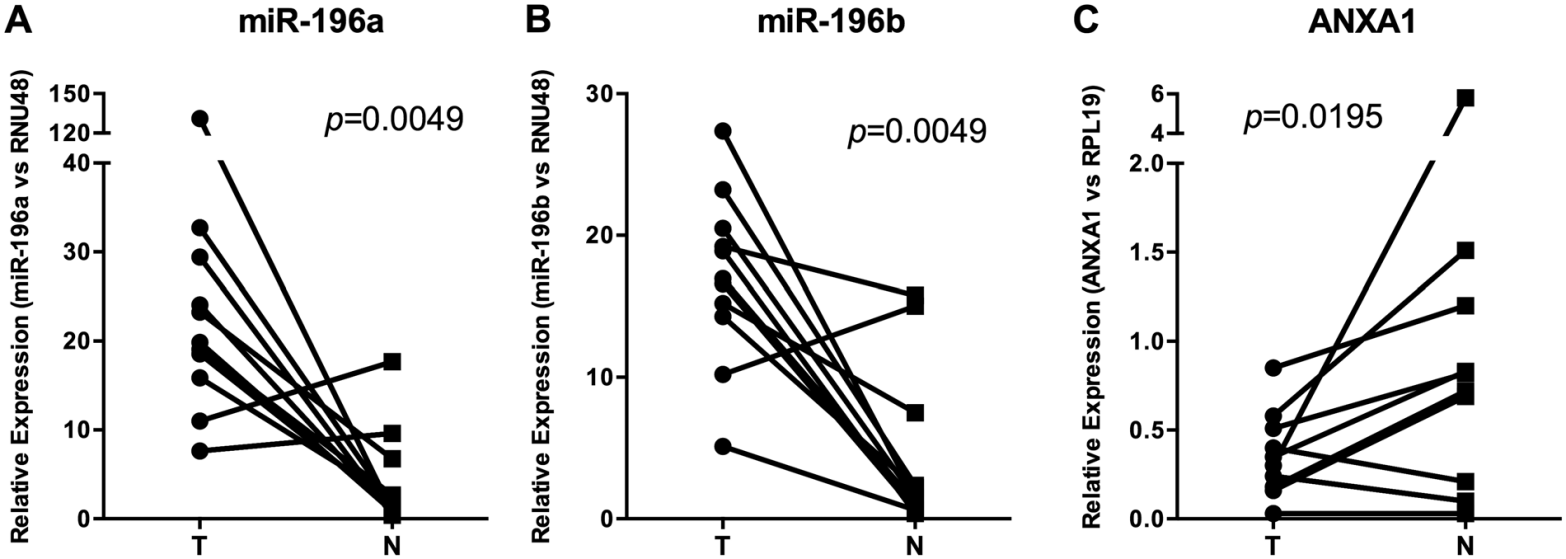
Analysis of miR-196a and miR-196b expression and ANXA1 mRNA levels in HNSCC tissue specimens. (**A**) miR-196a and (**B**) miR-196b expression levels were quantified by RT-qPCR in 11 fresh primary tumors (T) and patient-matched normal epithelia (N). Data were normalized to RNU48 levels, and relative to the normal mucosa from non-oncologic patients. (**C**) ANXA1 mRNA levels were quantified by RT-qPCR and data normalized to RPL19 levels. *p*-values obtained from the Wilcoxon test provided statistical confirmation of the significant changes.

miR-196a and miR-196b expression levels were analyzed using Taqman miRNA assays in 11 paired HNSCC tissue samples and patient-matched normal epithelia. Quantitative analysis of miR-196a and miR-196b expression showed increased levels of miR-196a and miR-196b in all tumors compared to normal epithelia from non-oncologic patients (Fig. 3A and B). Increased miR-196a and miR-196b levels were also detected in three patient-matched normal epithelia. Concomitantly, ANXA1 mRNA levels were significantly reduced in the tumor samples compared to paired normal epithelia (Fig. 3C). Consequently, the inverse correlation of ANXA1 mRNA levels with both miR-196a and miR-196b levels supports a role of miR-196a/b in the regulation of ANXA1 expression in HNSCC.

### ANXA1 is a direct target of both miR-196a and miR-196b in HNSCC-derived cell lines

**Figure 4:**
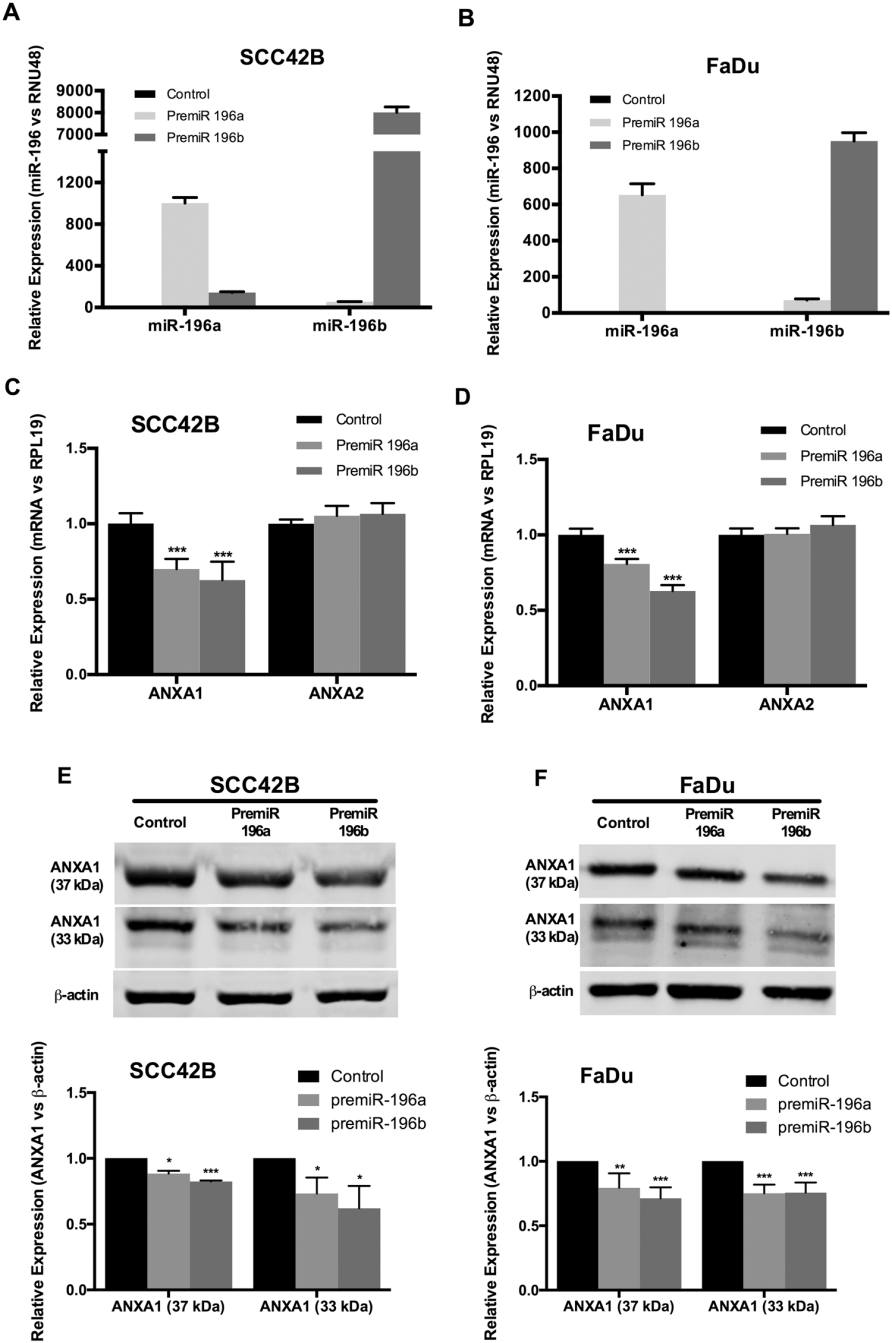
miR-196a and miR-196b specifically targeted ANXA1 expression in HNSCC-derived cell lines. miR-196a and miR-196b expression levels were quantified by RT-qPCR in SCC42B (**A**) and FaDu (**B**) cells transfected with either premiR-196a, premiR-196b or non-targeting control. Data were normalized to RNU48 levels and relative to control-transfected cells. mRNA expression levels of ANXA1 and ANXA2 were measured by qRT-PCR in SCC42B (**C**), and FaDu (**D**) after 72 h transfection with either premiR-196a, premiR-196b or non-targeting control. Data were normalized to RPL19 levels and relative to control-transfected cells. The graphs represent the mean ± SD, calculated from at least three independent experiments performed in triplicate. ANXA1 protein expression was analyzed by Western blot in SCC42B (**E**) and FaDu (**F**) cells after 72 h transfection with either premiR-196a, premiR-196b or non-targeting control. The graphs represent the mean ± SD, calculated from at least three independent experiments. *p < 0.05, **p < 0.01 and ***p < 0.001 by Student t-test.

We verified whether miR-196a and miR-196b regulated the endogenous expression of ANXA1 in HNSCC-derived cell lines. Transient transfection with specific pre-miR precursors pre-miR196a and pre-miR196b led to a robust induction of mature miR-196a and miR-196b levels (Fig. 4A and B). This was consistently accompanied by a significant inhibition of ANXA1 mRNA levels in both SCC42B and FaDu cells, while ANXA2 levels remained unaffected by pre-miR transfection (Figure 4C and D). ANXA1 protein expression was also down-regulated upon miR-196a and miR-196b overexpression in HNSCC cells both the full-length ANXA1 protein (37 kDa) and the cleaved form (33 kDa) (Fig. 4E and F).

**Figure 5:**
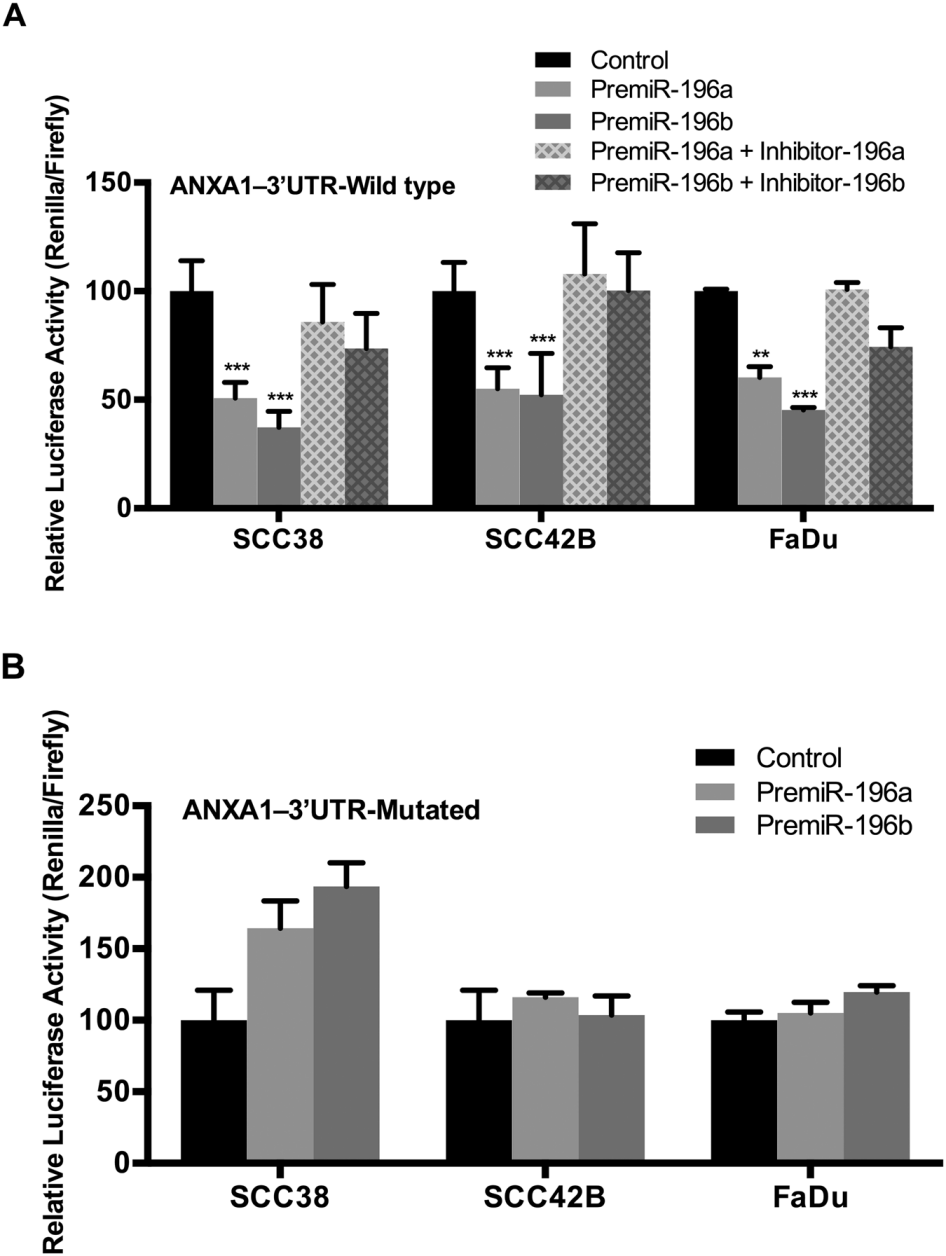
ANXA1 is directly targeted by both miR-196a and miR-196b in HNSCC cells. The constructs psi-CHECK-2-ANXA1–3′UTR-wt (**A**) and psi-CHECK-2-ANXA1–3′UTR-mut (**B**) were co-transfected with the indicated specific premiR precursors and miRNA inhibitors into SCC38, SCC42B, and FaDu cells. *Renilla* luciferase activity under the control of ANXA1 3′-UTR was measured and normalized to firefly luciferase, relative to control-transfected cells. The graphs represent the mean percentage ± SD, calculated from at least three independent experiments performed in quadruplicate. *p < 0.05, **p < 0.01 and ***p < 0.001 by Student t-test.

Consistent with these findings, we observed that luciferase reporter carrying ANXA1 3′-UTR region was sensitive to miR-196a and miR-196b ectopic expression (Fig. 5A). Thus, transfection with pre-mir196a and pre-mir196b led to a significant repression (approx. 50%) in all three HNSCC cell lines, while this effect was reverted by transfection with specific miR-196a and miR-196b inhibitors (Fig. 5A). In contrast, mutations of a miR-196a/b complementary site within ANXA1 3′-UTR prevented miR-196a/b-dependent repression (Fig. 5B). These results demonstrate that both miR-196a and miR-196b inhibited ANXA1 expression by its specific binding to the 3′-UTR region of ANXA1 mRNA. These data corroborate similar studies affirming that ANXA1 is a direct binding target of miR-196a^15, 22, 23^ and, more importantly, provide the first experimental evidence to prove that ANXA1 is a direct target of miR-196b.

### Analysis of *ANXA1* promoter activity in HNSCC-derived cell lines

**Figure 6:**
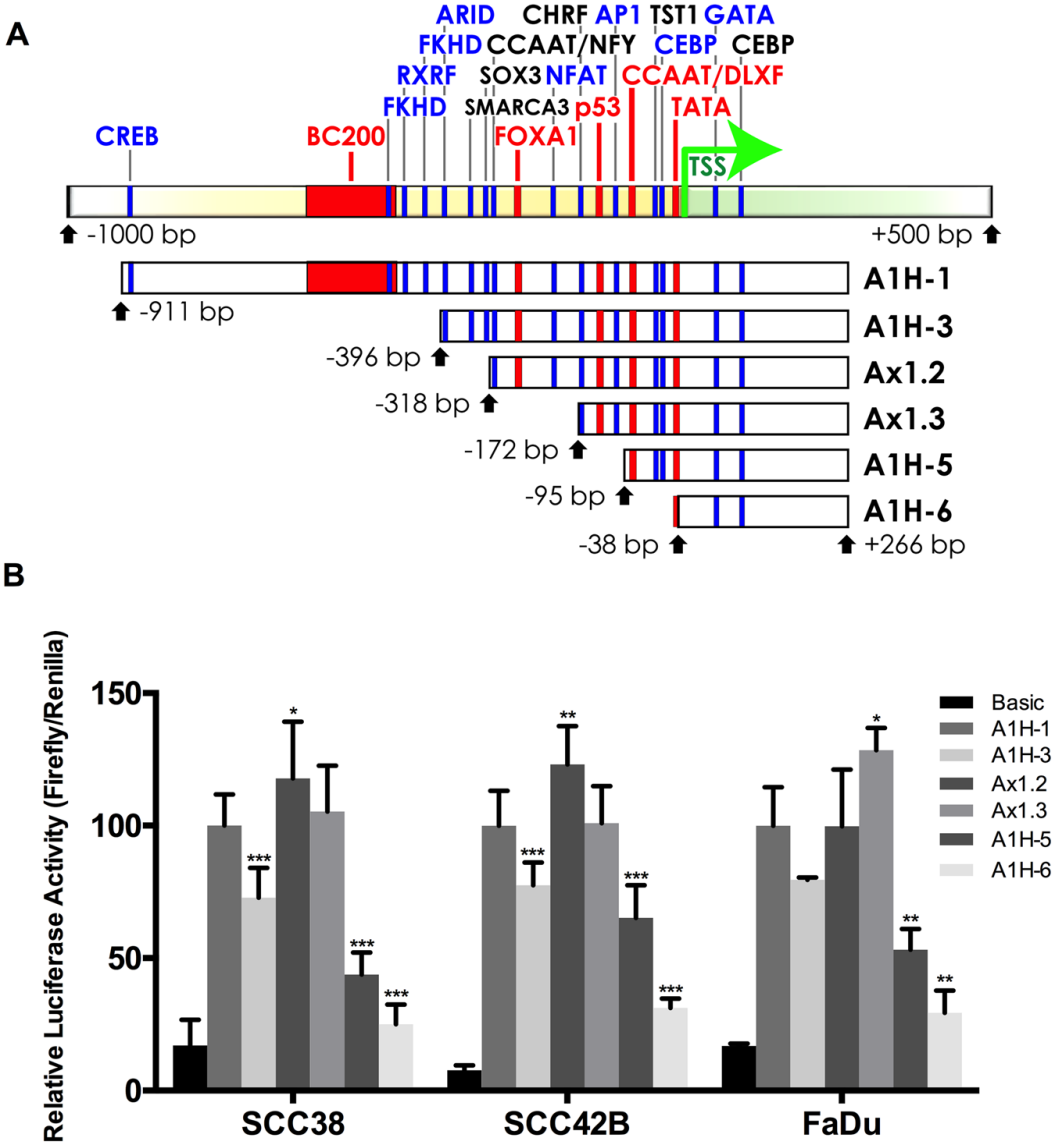
Deletion mapping of the promoter activity in the 5′-UTR sequence of *ANXA1* gene. (A) Schematic representation of the different *ANXA1* promoter fragments used in luciferase activity assays. The predicted binding sites for transcription factors relative to the transcription start site (TSS, +1) are also mapped. Promoter construct fragments of the proximal regulatory region (−1000 to +500 bp) of the human Annexin A1 gene (*ANXA1*), shown schematically in the six lower bars, were utilized in luciferase assays. The transcription start site (TSS) is marked by an arrow and other key elements (TATA-BP, CCAAT-box, p53, ForkHead/FOX) have been confirmed by high-throughput ChIP Seq studies (27, UCSC genome browser). The BC200 element^25^ may be a ncRNA with a role in squamous cell cancers^44^. Binding sites for other putative transcription factors were those predicted with highest confidence (>90%) by MatInspector (Genomatix). (**B**) Promoter activity of the A1H-1 to A1H-6 constructs in three different HNSCC-derived cell lines (SCC38, SCC42B and FaDu) measured with dual luciferase assays 48 h after transfection. Luciferase (firefly) activity was normalized to *Renilla* luciferase. The graphs represent the mean ± SD, calculated from at least three independent experiments performed in quadruplicate. *p < 0.05, **p < 0.01 and ***p < 0.001 by Student t-test.

We next assessed the *ANXA1* promoter activity in HNSCC cells using a series of deletion constructs from the 5′ *ANXA1* promoter region subcloned into the luciferase reporter plasmid pGL3-Basic. Figure 6 shows a schematic representation of the 5′ promoter fragments, including bioinformatic prediction of transcription factor binding sites in the *ANXA1* promoter sequence. The 5′ UTR *ANXA1* constructs controlling the expression of firefly luciferase were transiently transfected together with a control *Renilla* luciferase plasmid into three different HNSCC-derived cell lines SCC38, SCC42B and FaDu.

The activity of A1H-1 promoter fragment was robustly increased 5- to 15-fold in all three HNSCC cell lines compared to the pGL3-basic empty vector (Fig. 6). Promoter activity slightly decreased in all HNSCC cells when the −911/−396 region was deleted (A1H-3). Luciferase activity was further increased by deletion of the −396/−172 region (Ax1.2 and Ax1.3) in all three HNSCC cell lines, which suggested the existence of repressor elements within this region. Deletion of the −172/−95 sequence (A1H-5) containing the putative p53 binding site consistently and significantly decreased promoter activity in HNSCC cells. In addition, removal of −95/−38 sequence (A1H-6) containing a CCAAT box yielded to a further reduction in luciferase activity. According to these data, the −172/−38 region containing binding sites for p53 and AP1 and the CCAAT proximal box appear to be involved in the transcriptional regulation of ANXA1 expression in HNSCC cells.

## Discussion

Complex regulatory mechanisms pose a formidable challenge for deciphering gene expression patterns controlled by protein transcription factors, non-coding RNAs and their structural modifications at the post-transcriptional and epigenetic levels. A knowledge of the network components involved and their specific contribution to individual features of different cancers is a long-term therapeutic goal. We focused on the well-documented loss of ANXA1 protein expression in HNSCC^14, 15, 24^ by visualizing the changes using immunofluorescence microscopy. This technique vividly highlighted the progressive reduction from normal epithelia (including their nuclei) to precancerous lesions and ultimately to major loss in poorly differentiated tumors (Fig. 2). Detailed images of annexin A1 mRNA expression from *in situ* hybridization (Fig. 1) coincided with these protein changes supporting the hypothesis of a transcription-mediated mechanism. Only one previous microscopic study has demonstrated the concomitant changes in annexin A1 mRNA and protein levels in esophageal SCC^24^ and our results were complemented by RT-qPCR analysis of mRNA levels (Fig. 3) in accordance with previous studies^25, 26^. The earlier discovery that miR-196a specifically inhibited *Anxa1* mRNA translation by directly hybridizing to its 3′untranslated region argued for a post-transcriptional mechanism of mRNA destabilization in ESCC^15^, but this alone would not account for the eventual, complete loss of *Anxa1* nuclear mRNA observed here. Since it has been amply demonstrated that ANXA1 is a functional target of miR-196a in various cancers including HNSCC^15, 25, 26^, it remained necessary to determine the specificity and causal relationship between annexin A1 mRNA and protein levels with miR-196a and miR-196b expression.

The prediction by TargetScan^20^ that mature miR-196a1, a2 and b were candidates for binding *Anxa1* 3′UTR via sequence hybridization could conceivably account for the RNA degradation and consequent reduction in translation to ANXA1. However, both ANXA1 and miR-196 also target other genes including transcription factors that could modify their respective promoters to affect transcriptional activity. Thus, this inverse relationship is specific but not unique and the qualitative and quantitative changes may vary between tissues and cancer types^9–18^. The comparison of paired HNSCC tissue samples and patient-matched normal mucosa pointed to a fundamental regulatory control of ANXA1 by miR-196a/b in HNSCC, while *in vitro* studies established specificity for ANXA1, leaving ANXA2 unchanged, and the reproducibility in different cell types (Fig. 4). Even though the differences in ANXA1 protein levels following pre-miR196a overexpression were marginal, pre-miR196b overexpression consistently led to a significant reduction of ANXA1 expression at both mRNA and protein levels in both full-length (37 kDa) and cleaved form (33 kDa) in the two different HNSCC cell lines tested. In addition, down-regulation of ANXA1 protein was accompanied by a concomitant reduction in mRNA levels upon pre-miR196a and pre-miR196b overexpression. Subsequent direct analysis of various cell types (Fig. 5) provided further evidence that the *Anxa1* 3′untranslated region is a direct interaction target of miR-196a/b. This does not exclude the targeting of other gene products with a potential role in the transcriptional repression of *ANXA1* promoter activity and independent effects on the cancer phenotype. The inverse regulation between ANXA1 and miR-196 could also be mediated by induced changes in distinct miRNAs and relevant transcription factors. It is particularly noteworthy that ANXA1 and miR-196a have been shown to coregulate each other in a negative feedback loop in breast cancer through c-myc and NFkappaB^11^ and that ANXA1 regulation of other microRNAs and transcription factors could further contribute to the accompanying changes in NFkappaB and miR-196^10^.

An alternative scenario for esophageal SCC was recently presented^26^ in which ANXA1 induction of Snail and reduction of both E-cadherin and miR-196a reproduced the pathogenic phenotype, while miR-196a transfection had the opposite effect. miR-196b is a post-transcriptional inhibitor of the GATA transcription factor^27^, which interacts with ANXA1^28^ possibly by binding to its promoter (see Fig. 6a). Since mutations that affect the binding site interactions of miRNAs and their target genes can be relevant to tumorigenesis, our inclusion of the nucleotide variant miR-196b, located on a different chromosome than miR-196a, demonstrated qualitatively similar effects on ANXA1 and, by inference, binding affinity. The mechanisms of miR-196a/b transcriptional regulation and the possible roles of their respective HOX gene clusters as well as ANXA1 merit further investigation^10, 11^. The study of *ANXA1* promoter constructs in HNSCC-derived cell lines also established the presence of active regulatory sites in the 5′ non-transcribed region (−172 to −38 bp) (Fig. 6) with potential binding sites for transcription factors such as FOX, NFAT, p53, AP1, Sp1, CCAT, CEBP, TAT, GATA, corroborating and extending previous studies^29, 30^. Transcription factor binding to the p53 binding site or the CCAAT proximal box influenced by histone acetylation^30^ could also affect *ANXA1* transcriptional expression and possible tumor suppressor activity.

Our combined results showed that ANXA1 expression is markedly suppressed in all primary HNSCC tumors compared to the corresponding normal epithelia at the levels of both mRNA and protein. This coincided with an inverse regulatory role by miR-196a/b and ANXA1, presumably via direct effects on mRNA stability and protein translation, but conceivably also through secondary mediators of transcriptional control such as trans-acting protein factors. The question of sequential order thus becomes important to establish whether ANXA1 or miR-196a/b is primarily responsible for either initiating or promoting the pathogenic phenotype. Although we find that upstream changes in miR-196 can alter annexin A1 mRNA levels and hence protein levels, the idea that miR-196 is acting as regulator and ANXA1 as effector is overly simplistic in view of evidence for accompanying changes in many other signaling factors. Clearly, miR-196 and ANXA1 actions are part of a more complex regulatory network. Numerous other parameters such as transcription factors and miRNAs mentioned above may also be influenced secondarily by post-transcriptional modifications and epigenetic alterations and elucidation of the entire chain of events awaits more detailed scrutiny.

It has become apparent that miR-196a/b are altered in many forms of cancer^15, 31–34^ and are considered to have an oncogenic profile^25, 35–39^. The downstream inhibition of ANXA1 expression in turn has phenotypic consequences consistent with many of the known actions of ANXA1, including cell differentiation, proliferation, migration, apoptosis, immunosuppression and even vascular changes^1–5, 40^. The observations that miR-196 overexpression and annexin A1 underexpression are under both transcriptional and translational control would seem to offer a pathogenic mechanism in HNSCC. However, the opposing qualitative changes are dependent on the cancer type being considered^4, 18, 22^ and other effectors must contribute to the phenotypic consequences. The bidirectional regulation of miR-196a/b and ANXA1 could even represent a compensatory, homeostatic response to cancer progression^31^ influenced by the specific tumor microenvironment. Additional factors responsible for changes in miR-196a/b and ANXA1 thus merit further investigation to delineate the network of cellular processes responsible for initiating, promoting or suppressing oncogenesis in HNSCC^16, 18^. Our tentative conclusion is that ANXA1 and miR-196 changes are more closely related to the pathogenic features rather than the etiology of HNSCC and that correction of their misregulation could ameliorate conditions through cancer stem cell differentiation, its associated markers and decreased metastatic potential. Bioinformatics based approaches^41^ utilizing resources such as the Human miRNA Disease Database (HMDD) associations (http://www.cuilab.cn/hmdd) should help to gain further insight into the molecular mechanisms of HNSCC.

## Methods

### Patients and Tissue Specimens

Surgical tissue specimens (fresh frozen) from 11 patients with HNSCC who underwent resection of their tumors at the Hospital Universitario Central de Asturias between 2000 and 2002 were collected, in accordance to approved institutional review board guidelines. The clinicopathologic characteristics of these patients are shown in Supplementary Table S1. Patient-matched normal adjacent epithelia were also collected from all the patients. In addition, normal epithelia from non-oncologic patients without exposure to tobacco carcinogens (i.e. childhood tonsillectomy) were collected and used as healthy controls.

Representative tissue sections from HNSCC (FFPE) were obtained from archival, paraffin-embedded blocks from the Hospital Universitario Central de Asturias, in accordance to approved institutional review board guidelines. All experimental protocols were approved by the Institutional Ethics Committee of the Hospital Universitario Central de Asturias and by the Regional CEIC from Principado de Asturias. Informed consent was obtained from all patients. All patients had a single primary tumor (in the larynx or pharynx), microscopically clear surgical margins and received no treatment prior to surgery. In addition, FFPE tissue sections from patients with a diagnosis of laryngeal precancerous lesion (dysplasia) were obtained from the Hospital pathology archives. All the tissue sections selected also included normal adjacent epithelia as internal control.

### *In situ* hybridization

*In situ* hybridization was carried out following the procedure that we previously described^42^. The formalin-fixed paraffin-embedded tissues were cut into 4 µm sections onto Superfrost plus glass slides (Menzel-Glaser). The sections were deparaffinized, hydrated and rinsed in PBS. After pre-treatment with proteinase K (20 µg/ml) for 7.5 min at room temperature, the sections were rinsed in PBS, refixed with 3.7% paraformaldehyde and rinsed again in PBS. The slides were incubated with the labeled antisense *Anxa1* probes in the hybridization mix for 12 h at 60 °C. Subsequently, the sections were washed in 5x SSC, treated with 2x SSC containing 25% formamide, followed by sequential washes in 2x SCC, 0.2x SCC and rinsed in PBS-0.1% Tween 20 (PBT).

The *Anxa1* probe comprises the full coding cDNA sequence obtained by RT-PCR from human placenta RNA and subcloned in pBSK(SK+ ). Digoxigenin-labeled antisense and sense riboprobes were prepared by using this plasmid and DIG-UTP for *in vitro* transcription reactions, with T3 and T7 RNA polymerases using the DIG-RNA labeling kit T3/T7 (Roche). For the hybrid detection of digoxigenin antibody, the samples were pre-blocked with 5% sheep serum in PBS-T for 30 min and then incubated with anti-digoxigenin antibody conjugated with alkaline phosphatase (Roche) diluted 1:2000 in PBT containing 5% sheep serum overnight at 4 °C and visualized with NBT-system (Roche). The tissues were counterstained with nuclear red for 5 min as the final step. Following staining, the slides were dehydrated through graded alcohols and mounted with the coverslip. The counterstained sections were evaluated with a Zeiss Axioscope 2 Plus microscope, photographed with Zeiss Axio-Cam HRC camera, and images were rendered by Adobe Photoshop software.

### Immunofluorescence

The formalin-fixed paraffin-embedded tissues were cut into 4 µm sections onto Superfrost plus glass slides (Menzel-Glaser). The sections were deparaffinized with standard xylene and hydrated through graded alcohols into water. While hydrating the deparaffinized sections in graded alcohol, the slides were immersed for 1 h in 70% ethanol supplemented with 0.25% NH_3_, and rehydration was resumed by immersion in 50% ethanol for 10 min and rinsed in PBS. Before immunostaining, the samples were sequentially incubated in 0.5% Triton X-100 in PBS for 30 min, 0.1 M glycine in PBS for 15 min, and blocked with 0.1% bovine serum albumin in PBS for 30 min. They were rinsed in PBS-0.05% Tween 20, incubated for 2 h with mouse anti-Annexin A1 monoclonal antibody (BD Transduction Laboratories) diluted in PBS, washed in PBS containing 0.05% Tween 20, and incubated for 45 min with the appropriate secondary antibodies conjugated to fluorescein isothiocyanate (FITC) (Jackson Immunoresearch Laboratories, West Grove, PA). The samples were counterstained with Propidium Iodide (PI) for the cytochemical demonstration of nucleic acids. Finally, the coverslips were mounted in VectaShield (Vector Laboratories, Peterborough, United Kingdom) and sealed with nail polish. Confocal microscopy was performed with a Leyca TSC-SP2-AOBS laser confocal microscope, using wavelengths of 488 nm (for FITC) and 543 nm (for IP). Each channel was recorded independently, and pseudocolor images were generated and superimposed. Images were handled by the Adobe Photoshop 7.0 software.

### miRNA expression analysis

Total RNA (including miRNA) was extracted from fresh frozen tissue and/or cultured cells using Trizol (Invitrogen Life Technologies). The relative levels of miR-196a and miR-196b were determined by two-step RT-PCR using miRNA-specific primers and probes according to the Taqman miRNA Assay protocol (Applied Biosystems) on a StepOnePlus Real-Time PCR System (Applied Biosystems). Reactions were run in triplicate, using RNU48 as control. The relative miRNA expression was calculated using the 2^−ΔΔ*C*T^ method and the data were normalized to RNU48 levels and relative to the normal mucosa from non-oncologic patients.

### Cell culture

The HNSCC-derived cell lines SCC38 and SCC42B were kindly provided by Dr. R. Grenman (Department of Otolaryngology, University Central Hospital, Turku, Finland)^43^. FaDu cells were purchased to the ATCC. Cells were grown in DMEM supplemented with 10% fetal bovine serum (FBS), 100 U/ml penicillin, 200 mg/ml streptomycin, 2 mmol/l L-glutamine, 20 mmol/l HEPES (pH 7.3) and 100 mmol/l non-essential amino acids.

### Transfections with pre-miR miRNA precursors

Ambion Pre-miR miRNA Precursors AM17100 hsa-miR-196a (#PM10068) and hsa-miR-196b (#PM12946) were purchased to Thermo Fisher Scientific and siGLO RISC-Free™ was used as non-targeting control (Thermo Scientific Dharmacon).

HNSCC cells were seeded in 6-well plates at a density of 60,000 cells. The next day, cells were transfected with 100 nM of either pre-miR196a or pre-miR196b or a non-targeting control using Lipofectamine 2000 Transfection Reagent (Invitrogen Life Technologies), according to the manufacturer’s protocol. miRNA expression or mRNA gene expression was tested by real-time RT-PCR at 72 h post-transfection.

### RNA extraction and real-time RT-PCR

Total RNA was extracted from HNSCC cells using Trizol reagent (Invitrogen Life Technologies), and cDNA synthesized with Superscript II RT-PCR System (Invitrogen Life Technologies), according to manufacturer’s protocols. Gene expression was analyzed by Real-time PCR using the StepOnePlus Real-Time PCR System (Applied Biosystems) following Applied Biosystems’ SYBR Green Master Mix protocol. Reactions were carried out using the primers detailed in Supplementary Table S2. The constitutively expressed RPL19 ribosomal coding gene was used as endogenous control. The relative mRNA expression was calculated using the 2^−ΔΔ*C*T^ method and the data were expressed as the fold-change normalized to RPL19 mRNA levels and relative to normal epithelia from oncologic patients (or control-transfected cells).

### Western blot analysis

Proteins were extracted from HNSCC cells transfected with either pre-miR196a, pre-miR196b or a non-targeting control at 72 h post-transfection using lysis buffer (R&D Systems). Protein lysates were resolved by SDS–polyacrylamide gel electrophoresis (SDS–PAGE) and transferred subsequently to nitrocellulose membranes (Amersham Protran, GE Healthcare).

The membranes were blocked for 1 h with Odyssey blocking buffer and incubated overnight with rabbit polyclonal ANXA1 (at 1:1,000 dilution) raised in our laboratory against the human recombinant protein and the anti-β-actin (dilution 1:10,000 for 1 h; from Sigma Aldrich # AC15). The IRDye Infrared Fluorescent secondary antibodies Goat anti-Rabbit IRDye 800CW and Goat anti-Mouse IRDye 680RD were used for detection. Membranes were scanned with the Odyssey Fc Dual-Mode Imaging System (LI-COR Biosciences) using the red (700 nm) and green (800 nm) channels.

### Luciferase reporter assays

Dr. Jacques Huot kindly provided the constructs psi-CHECK-2-ANXA1–3′UTR-wt containing *Renilla* luciferase under the control of ANXA1 3′-UTR and firefly luciferase as a control and psiCHECK-2-ANXA1–3′UTR-mut generated by directed mutagenesis in the predicted miR196 binding site^22^.

HNSCC cells were seeded into 96-well plates in antibiotic-free medium at a density of 4,000 cells *per* well (6,000 cells for SCC38). The next day, cells were transfected with psi-CHECK-2-ANXA1–3′UTR-wild type or psiCHECK-2-ANXA1–3′UTR-mutated (20 ng/well) together with pre-miR196a or pre-miR196b or a non-targeting control (100 nM) or miRNA inhibitors using Lipofectamine 2000. Cells were lysed 48 h after transfection and luciferase activity evaluated using Dual-Luciferase Reporter Assay System following manufacturer’s protocol (Promega). *Renilla* luciferase activity which is under the control 3′-UTR insert was evaluated and firefly luciferase was measured as a loading control in each condition using a Synergy HT Multi-Mode Microplate Reader (Biotek).

For *ANXA1* promoter analysis, several plasmid constructs, containing different fragments of the promoter region of *ANXA1* gene, were obtained. The construct A1H-1 (−911/+266) was made by PCR amplification with specific oligonucleotide primers and a 3633 bp *Xba*I subclone spanning from −2206 to +1427 positions^44^ as template. The resulting 1177 bp PCR product was blunt ended and subcloned into the *Sma*I site of pBSK plasmid and sequenced to determine orientation. From this plasmid a fragment excised with *Stu*I and *Hin*d3 was purified and ligated into the *Sma*I and *Hin*dIII sites of pGL3 Basic vector (Promega). Similarly for the A1H-3 (−396/+266), a previous genomic subclone was generated in pBSK vector containing the desired fragment spaning from the *Eco*RI site at position −396 to +266 which was subsequently excised also with *Sma*I and *Hin*dIII, purified and ligated into the *Sma*I and *Hin*dIII sites of pGL3 Basic vector (Promega). Other constructs Ax1.2 (−318/+266) and Ax1.3 (−172/+266) were kindly provided by Dr. SE Moss^29^, and A1H-5 (−95/+266) and A1H-6 (−38/+266) by Dr. MA Lizarbe^30^.

24 h before transfection, HNSCC cells were seeded into 96-well plates in complete media at a density of 4,000 cells *per* well (6,000 cells for SCC38). Cells were transfected using FuGENE 6 (Promega) with each of the indicated *ANXA1* reporter constructs (30 ng/well) or the empty vector pGL3-Luc (Promega) and the pRL-TK (Promega) control plasmid (10 ng). The pSG5 empty vector was used to normalize the total DNA amount to 50 ng *per* well. After 48 h transfection, cells were lysed and extracts assayed for luciferase activity using Dual-Luciferase Reporter Assay System (Promega). Firefly luciferase activity was normalized to *Renilla* activity.

### Statistical analysis

The data are presented as the mean ± standard deviation (SD), and compared using Student’s t-test. Wilcoxon test was used for paired samples. Statistical analysis was performed using GraphPad Prism version 6.0 (Graphpad Software Inc, La Jolla, CA, USA). *p* values less than 0.05 were considered statistically significant (*p* < 0.05, *; *p* < 0.01, **; *p* < 0.005, ***).

## Electronic supplementary material

Supplementary Information
