# Supplementary material for: Annexin A1 down-regulation in head and neck squamous cell carcinoma is mediated via transcriptional control with direct involvement of miR-196a/b
Source: Sci Rep. 2017 Jul 28;7:6790. doi: 10.1038/s41598-017-07169-w (PMC5533727; doi:10.1038/s41598-017-07169-w)
Supplement: Supplementary file 1 — Supplementary Information [file 41598_2017_7169_MOESM1_ESM.pdf]

SUPPLEMENTARY INFORMATION

Title: Annexin A1 down-regulation in head and neck squamous cell carcinoma is mediated via transcriptional control with direct involvement of miR-196a/b

AUTHORS:

Saúl Álvarez-Teijeiro[1], Sofía T. Menéndez[1], M. Ángeles Villaronga[1], Emma Pena-Alonso[1], Juan P. Rodrigo[1], Reginald O. Morgan[2], Rocío Granda-Díaz[1], Cecilia Salom[1], M. Pilar Fernandez[2*], Juana M. García-Pedrero[1*].

[1] Department of Otolaryngology, Hospital Universitario Central de Asturias and Instituto Universitario de Oncología del Principado de Asturias, University of Oviedo, Oviedo, CIBERONC, Spain.

[2] Department of Biochemistry and Molecular Biology, University of Oviedo, Oviedo, Spain.

* ***Correspondence***:

Juana María García-Pedrero, PhD E-mail: juanagp.finba@gmail.com

Hospital Universitario Central de Asturias, Edificio FINBA, Lab ORL, Avda Roma s/n 33011 Oviedo, Spain

M. Pilar Fernandez, PhD E-mail: pfernandez@uniovi.es

Departamento de Bioquímica. Edificio Santiago Gascón, Campus del Cristo, 33006 Oviedo, Spain

Supplementary Table S1

Clinicopathologic characteristics of HNSCC patients

Case	Sex	Age (yrs)	Tobacco	Alcohol	Site	pT Class	pN Class	Histologic differentiation
1	Male	66	50 PY	Heavy	Oral Cavity	4	2	Well
2	Male	66	60 PY	Heavy	Oropharynx	3	0	Poor
3	Male	38	30 PY	Heavy	Oropharynx	4	0	Well
4	Male	64	20 PY	Heavy	Oropharynx	4	3	Moderate
5	Male	80	60 PY	Mild	Larynx	4	0	Well
6	Male	61	40 PY	Moderate	Hypopharynx	3	2	Moderate
7	Male	70	40 PY	Mild	Larynx	2	0	Well
8	Male	66	45 PY	Mild	Larynx	1	0	Well
9	Male	77	35 PY	Mild	Oropharynx	1	3	Poor
10	Male	52	30 PY	Mild	Larynx	2	0	Well
11	Male	58	50 PY	Heavy	Oropharynx	4	3	Poor

PY: Packs-year. Alcohol: Mild, <50 gr/day; Moderate, 50-100 gr/day; Heavy, >100 gr/day

Supplementary Table S2

Primers used for real-time RT-PCR (5’ → 3’)

ANXA1-Fw	GCAGGCCTGGTTTATTGAAA
ANXA1-Rv	GCTGTGCATTGTTTCGCTTA
ANXA2-Fw	CTCTACACCCCCAAGTGCAT
ANXA2-Rv	TCAGTGCTGATGCAAGTTCC
RPL19-Fw	GCGGAAGGGTACAGCCAAT
RPL19-Rv	GCAGCCGGCGCAAA
